# Role of toll-like receptors and nod-like receptors in acute lung infection

**DOI:** 10.3389/fimmu.2023.1249098

**Published:** 2023-08-16

**Authors:** John Le, Yathushigan Kulatheepan, Samithamby Jeyaseelan

**Affiliations:** ^1^ Laboratory of Lung Biology, Department of Pathobiological Sciences and Center for Lung Biology and Disease, School of Veterinary Medicine, Louisiana State University (LSU) and Agricultural & Mechanical College, Baton Rouge, LA, United States; ^2^ Section of Pulmonary and Critical Care Department of Medicine, LSU Health Sciences Center, New Orleans, LA, United States

**Keywords:** TLR - toll-like receptor, NOD (nucleotide binding and oligomerization domain) and leucine rich repeat containing receptor (NLR), lung, Neutrophil, cytokine

## Abstract

The respiratory system exposed to microorganisms continuously, and the pathogenicity of these microbes not only contingent on their virulence factors, but also the host’s immunity. A multifaceted innate immune mechanism exists in the respiratory tract to cope with microbial infections and to decrease tissue damage. The key cell types of the innate immune response are macrophages, neutrophils, dendritic cells, epithelial cells, and endothelial cells. Both the myeloid and structural cells of the respiratory system sense invading microorganisms through binding or activation of pathogen-associated molecular patterns (PAMPs) to pattern recognition receptors (PRRs), including Toll-like receptors (TLRs) and NOD-like receptors (NLRs). The recognition of microbes and subsequent activation of PRRs triggers a signaling cascade that leads to the activation of transcription factors, induction of cytokines/5chemokines, upregulation of cell adhesion molecules, recruitment of immune cells, and subsequent microbe clearance. Since numerous microbes resist antimicrobial agents and escape innate immune defenses, in the future, a comprehensive strategy consisting of newer vaccines and novel antimicrobials will be required to control microbial infections. This review summarizes key findings in the area of innate immune defense in response to acute microbial infections in the lung. Understanding the innate immune mechanisms is critical to design host-targeted immunotherapies to mitigate excessive inflammation while controlling microbial burden in tissues following lung infection.

## Introduction

Respiratory diseases constitute major health and financial burdens worldwide. In fact, five major respiratory diseases represent the most common causes of severe illness and death in humans (1). Of these five diseases, acute lower respiratory tract infections cause an more than 4 million deaths per year and are the leading cause of mortality in children 5 years or under (1). Pneumonia is an important clinical issue in both healthy and immunocompromised individuals and accounts for more than 800,000 hospitalizations in the United States annually (1–3). Furthermore, pneumonia is the predominant cause of mortality in children under 5 years of age (1). When respiratory infections overpower the host’s immunity, pneumonia is associated with widespread lung pathology by inducing excessive oxidative stress in the alveolar-capillary compartment (4). Pulmonary bacterial, viral, and fungal infections are major causes of infectious death in all age groups and are a key risk factor for Acute Lung Injury (ALI)/Acute Respiratory Distress Syndrome (ARDS), for which there are currently no therapies available (5–7). The innate immune response is consequential in effective host defense against and clearance of invading pathogens (5, 8). Lung epithelial cells are the first to encounter the pathogen in the lung during pneumonia and other lung infections, which is followed by an influx of neutrophils and macrophages to clear the pathogen (9). This is initiated in the mammalian immune system by pattern recognition receptors (PRR) that sense pathogen-associated molecular patterns (PAMPs) in order to produce proinflammatory responses (9).

**Figure 1:**
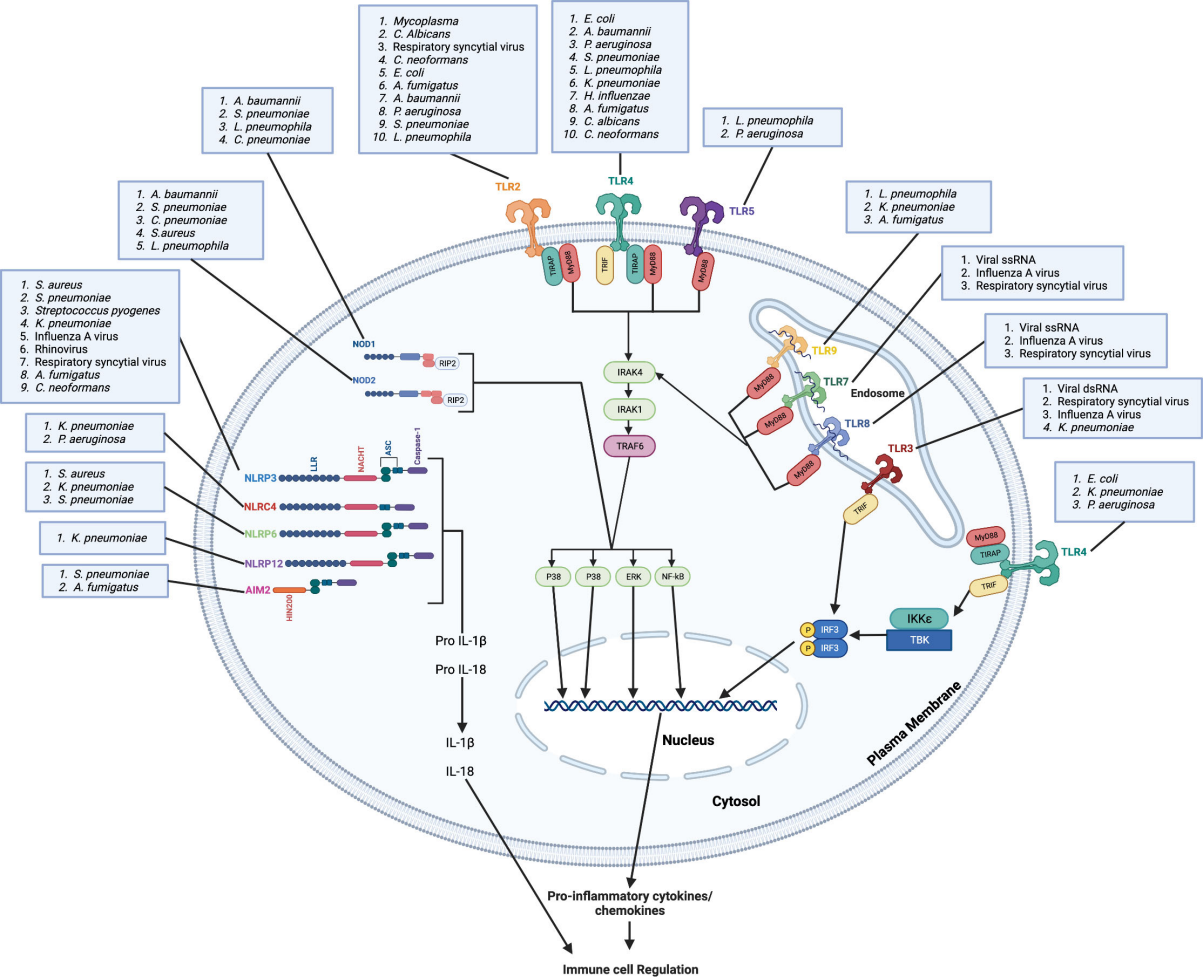
Microbes are recognized by membrane bound and cytoplasmic pattern recognition receptors. Plasma membrane-bound TLRs (TLR2, TLR4 and TLR5) and endosome membrane-bound TLRs (TLR3, TLR7, TLR8 and TLR9) recognize bacterial, viral, and fungal lung pathogens and/or PAMPs. TLR4, TLR5, TLR6, TLR7 and TLR9 recruit MyD88 directly to the TIR domain while TLR2 and TLR4 requires TIRAP for the recruitment of MyD88 to the TIR domain. TLR3 recruits TRIF to the TIR domain. Through the MyD88-independent pathway, TLR4 requires TRAM for the recruitment of TRIF. The binding of pathogens and/or PAMPs to TLRs leads to complex downstream signaling cascades that result in transcription of pro-inflammatory mediators and activation of MAP kinases. Cytosolic NOD1 and NOD2 recognize bacterial, viral, and fungal pathogens in the lung and mediate signaling by RIP2. The NLRP3, NLRP6, NLRP12 senses PAMPs using the LRR domain and uses ASC to recruit caspase-1 and induce the downstream signaling cascade, which result in transcription of pro-inflammatory mediators and activation of MAP kinases. The HIN200 domain of AIM2 binds cytoplasmic DNA and recruits ASC and caspase-1 to induce the downstream signaling cascade. NLRC4 does not require ASC to recruit caspase-1. These pro-inflammatory mediators, including chemokines, lead to the regulation of immune cell infiltration to the lung and the induction of inflammation. Created with Biorender.com.

PRRs can be categorized as membrane bound PRRs and cytoplasmic PRRs according to their cellular location. The first category comprises Toll-like receptors (TLRs) and C-type lectin receptors that survey the extracellular and endosomal locations for the presence of PAMPs, and the second category of PRRs contains nucleotide-oligomerization domain (NOD)-like receptors (NLRs) and retinoic acid-inducible gene-like receptors (RIG-I) that survey intracellular compartments for PAMPs (10). TLRs are expressed by a wide variety of immune cells, including macrophages, neutrophils, natural killer cells and dendritic cells, and are responsible for triggering a signaling cascade of proinflammatory responses against invading microbes (11). These receptors play a critical role in detecting and responding to the presence of microbes in the body (12). TLRs are activated by PAMPs that are unique to bacterial cell walls, such as lipopolysaccharides (LPS) present in Gram-negative bacteria, and peptidoglycans found in Gram-positive bacteria. Upon activation, TLRs trigger a signaling cascade that leads to the activation of numerous transcription factors, including NF-κB, which induce the upregulation of pro-inflammatory cytokines and chemokines, as well as other antimicrobial proteins (13, 14). So far, 23 NLRs have been identified in humans whereas 34 have been discovered in mice (15, 16). NLRs are multi-domain protein complexes comprising of a middle NOD (NOD or NACHT) domain flanked by C-terminal leucine-rich repeats (LRRs) that recognize PAMPs along with a variable N-terminal region containing either baculovirus inhibitor repeats (BIR) or a caspase activation and recruitment domain (CARD), a pyrin domain (PYD) (16). While NLRs are predominately expressed in cells of the innate immune system, such as neutrophils, macrophages, dendritic cells and endothelial cells, they can also be found in cells of the adaptive immune system (17). NLRs are unique because, unlike other classes of receptors, many NLRs can form supramolecular complexes, known as inflammasomes, by recruiting apoptosis-associated speck-like protein (ASC) and caspase-1 or -11 after recognition of certain PAMPs (18, 19). The formation of the inflammasome leads to the cleavage and activation of the caspase, and the activated caspase can then convert interleukin-1β (IL-1β) and IL-18 into their active forms to initiate inflammatory signaling (20). This review focuses on innate immune cascades involved in host defense against microbes, including bacterial, viral, and fungal pathogens (**Figure 1**).

## Pulmonary bacterial infection

While antibiotics have reduced the overall morbidity caused by bacterial pneumonia, the mortality rate among hospitalized patients remains significantly high, especially in elderly and immunocompromised populations (21, 22). In addition, the overuse of antibiotics to control pneumonia and other bacterial infections has resulted in the emergence of multiple antibiotic-resistant bacterial pathogens. Methicillin-Resistant *Staphylococcus aureus* (MRSA) is a Gram-positive bacterium that causes serious public health threats. USA300 is the most common MRSA strain and causes severe infections in children and adults (23, 24). Other common bacterial pathogens that are known to cause severe lung diseases include *Streptococcus pneumoniae* and *Klebsiella pneumoniae*. *Streptococcus pneumoniae*, a Gram-positive bacterium, is a significant human pathogen that causes a wide range of diseases including pneumonia, meningitis, and septicemia (25). Though pneumococcal diseases caused by pneumococcal serotype 2 strains are associated with lower mortality, pneumonia caused by pneumococcal serotype 3 strains is the most common and is associated with a higher risk of death in adults (25). *Klebsiella pneumoniae*, a Gram-negative bacterium, causes severe pneumonia with extensive parenchymal damage in the lungs. The spread of carbapenem-resistant *K. pneumoniae* strains is of particular concern, causing ≥50% mortality especially in patients with diabetes and in heavy alcohol consumers (26, 27). The emergence of these multidrug-resistant bacterial strains increases the necessity for alternative therapeutic options. The initial phase of bacterial infection in the lung is characterized by neutrophil-dependent inflammation (1, 4). While neutrophil-mediated inflammation helps in the elimination of bacteria, it also causes bystander parenchymal injury, and when excessive, this injury may lead to clinical ARDS (1, 4). Therefore, it is necessary to discover the molecular and cellular mechanisms that trigger lower respiratory tract infection and ALI/ARDS to create new therapeutic methods to improve host immune mechanisms to control microbial growth and multiplication while attenuating microbe-mediated parenchymal injury.

### TLRs

Microbial components engage with Toll-like receptors (TLRs) to initiate downstream signaling pathways and induce genes involved in host defense (**Table 1**). TLRs play a crucial role in the recognition and clearance of bacterial infections. TLR1 recognizes bacterial lipoproteins and is associated with the recognition of other Gram-positive bacteria (83, 84). TLR2 is not only essential for the recognition of peptidoglycan on the surface of Gram-positive bacteria, such as *S. pneumoniae*, but also recognizes bacterial lipoproteins, lipoteichoic acid and fungal cell wall components (33, 83, 85). TLR2 can also work in conjunction with TLR1 or TLR6 to recognize diacylated or triacylated bacterial lipoproteins, respectively (83, 86). Researchers have shown TLR2 recognizes the lipopolysaccharides of *Legionella pneumophila* and induces chemokine-dependent cellular migration that is crucial for the host innate response in *L. pneumophila-*induced pneumonia (30, 31). A recent study has shown the outer membrane vesicles (OMVs) released by *Acinetobacter baumannii*, a Gram-negative bacterium, trigger the activation of TLR2 and TLR4 and lead to the release of several proinflammatory chemokines and cytokines in the lungs of mice (28). However, a previous study has shown that TLR2 activation during *A. baumannii* infection decreases survival associated with lower neutrophil recruitment in the deficient mice than the wild-type (WT) controls (29). Though TLR3 is associated with double-stranded RNA (dsRNA) from viral infections, a recent study has shown TLR3 activation results in increased susceptibility and mortality in *K. pneumoniae*-induced pneumonic mice (36). TLR4 recognizes lipopolysaccharide (LPS), a component of the outer membrane of Gram-negative bacteria, such as *A. baumannii* (28), *Haemophilus influenzae* (39)*, K. pneumoniae* (40), and *Pseudomonas aeruginosa* (32). A recent study has shown that TLR4 uses the MyD88 signaling axis to regulate monocyte differentiation and neutrophil infiltration to increase survival and decrease bacterial burden in the lungs of mice infected with *S. pneumoniae* (41). TLR5 is a surface receptor that recognizes the bacterial flagellin protein (87). Researchers have shown TLR5 is involved in the induction of pulmonary defenses during infection with *P. aeruginosa* and *L. pneumophila* (42, 43). TLR9 recognizes unmethylated DNA with cytosine-phosphate-guanosine (CpG) motifs that are found in bacterial and viral DNA (88, 89) and regulates responses during common pulmonary bacterial infections such *L pneumophila* (49), *S. pneumoniae* (50), and *K. pneumoniae* (48). The function of TLR10 is not yet fully understood, but it may play a role in modulating the immune response (83).

**Table 1:** The role of innate immune molecules during acute microbial infections in the lung.

Phenotype	Infection	Survival	Neutrophil influx	Bacterial, viral, or fungal burden	Bacterial viral, or fungal dissemination
TLRs					
TLR2	*A. baumannii* (28, 29)	ND	↑	↓	ND
	*L. pneumophila* (30, 31)	↓	↓	↑	NS
	*P. aeruginosa* (32)	ND	NS	↓ early	ND
	*S. pneumoniae* (33)	↑	↓	NS	NS
	Respiratory syncytial virus (34)	ND	↓	ND	ND
	*A. fumigatus* (35)	NS	ND	↓	ND
TLR3	*K. pneumoniae* (36)	↑	↑	↓	↓
	Influenza A virus (37)	↑	↑	↓	↓
	Respiratory syncytial virus (38)	ND	ND	ND	ND
TLR4	*A. baumannii* (29)	ND	↓	↓	ND
	*H. influenzae* (39)	ND	↓	↑	ND
	*K. pneumoniae* (40)	↓	ND	↑	ND
	*P. aeruginosa* (32)	N	↓ late	NS	ND
	*S. pneumoniae* (41)	↓	↓	↑	ND
	*A. fumigatus* (35)	NS	ND	↑	ND
TLR5	*L. pneumophila* (42)	ND	↓ early	NS	ND
	*P. aeruginosa* (43)	↓	↓	↑	↑
TLR7	Influenza A virus (44)	↑	↑	NS	ND
	Respiratory syncytial virus (45)	ND	ND	ND	ND
TLR8	Influenza A virus (46)	ND	ND	ND	ND
	Respiratory syncytial virus (47)	ND	ND	ND	ND
TLR9	*K. pneumoniae* (48)	↓	ND	↑	↑
	*L. pneumophila* (49)	↓	NS	↑	ND
	*S. pneumoniae* (50)	↓	NS	↑	↑
TLR adaptors					
MyD88	*E. coli* (51)	↓	↓	ND	ND
	*H. influenzae* (39)	ND	ND	↑	ND
	*K. pneumoniae* (52)	↓	↓	↑	↑
	*L. pneumophila* (31)	ND	↓	↑	↑
	*P. aeruginosa* (53)	ND	↓	↑	↑
	*S. aureus* (53)	ND	↓	NS	NS
	*S. pneumoniae* (54)	↓	↓	↑	↑
	*A. fumigatus* (35)	NS	ND	↓	ND
TIRAP	*K. pneumoniae* (55)	↓	↓	↑	↑
	*E. coli* (56)	ND	↓	↑	ND
TRIF	*E. coli* (51)	↓	↓	↑	↑
	*P. aeruginosa* (57)	ND	↓	↑	ND
	*K. pneumoniae* (52)	↓	↓	↑	↑
NODs					
NOD1	*A. baumannii* (58)	ND	↓	↑	ND
	*C. pneumoniae* (59)	↓	↓	↑	ND
	*L. pneumophila* (60)	ND	↑	↑	ND
	*S. pneumoniae* (61, 62)	ND	ND	↓	ND
NOD2	*A. baumannii* (58)	ND	↓	↑	ND
	*S. aureus* (63)	↓	NS	↑	↑
	*C. pneumoniae* (59)	↓	↓	↑	ND
	*S. pneumoniae* (64)	ND	NS	↑ early	ND
	*L. pneumophila* (60)	ND	↑	NS	ND
NOD Adaptors					
RIP2	*C. pneumoniae* (59)	↓	↓ early	↑	ND
	*L. pneumophila* (65)	ND	↓	↑	ND
	*E. coli* (56)	ND	↓	↑	ND
NLRs					
NLRP3	*S. aureus* (66)	↓	↓	NS	ND
	*S. pneumoniae* (67)	↓	NS	NS	↑
	*K. pneumoniae* (68)	↓	↓	ND	ND
	Influenza virus (69)	ND	↓	ND	ND
	Respiratory syncytial virus (34)	ND	↓	ND	ND
	Rhinovirus (70)	ND	↓	ND	ND
	*A. fumigatus* (71)	↓	ND	ND	ND
	*C. neoformans* (72)	ND	↓	↑	ND
NLRC4/IPAF	*P. aeruginosa* (73)	↓	NS	↑	↑
	*K. pneumoniae* (74)	↓	↓	↑	↑
NLRP6	*S. aureus* (75)	↑	↑	↓	ND
	*S. pneumoniae* (76)	↑	↑	↓	ND
	*K. pneumoniae* (77)	↓	↓	↑	↑
NLRP12	*K. pneumoniae* (78)	↓	↓	↑	↑
NLR Adaptor					
ASC	*P. aeruginosa* (79)	NS	NS	ND	ND
	Influenza*/* *S. aureus* (80)	↓	↓	↓	ND
AIM2 Inflammasome	*S. pneumoniae* (81)	↓	ND	↑	ND
	*A. fumigatus* (82)	↓	ND	ND	ND

• Phenotype was determined by mainly using whole-body knockout or transgenic mice post-infection.

• ND, not determined; NS, no significant difference.

• Neutrophil influx was determined in BALF and/or lung parenchyma.

• Bacterial, viral, or fungal burden was measured in the lungs.

• Bacterial, viral, or fungal dissemination was measured in blood or spleen.↑ and ↓ represent change in expression compared to wild-type.

In general, TLRs are associated with five separate adaptor molecules (TRIF, MyD88, TIRAP, SARM, and TRAM), which are recruited to the cytoplasmic TIR domain of the TLRs (84, 90) (**Table 1**). MyD88 is essential for TLR2, TLR4, TLR5, TLR6, TLR7, and TLR9 and is recruited to the TIR domain (90). For TLR2 and TLR4, TIRAP is required for the recruitment of MyD88 and subsequent signaling (90). TLR3 and TLR4 signaling includes the MyD88-independent pathway, where TRIF plays an important role (91). TRAM is an important adaptor in TLR4 associated TRIF-mediated but MyD88-independent signaling (84, 90). The recruitment of MyD88 enables the association of IL-1R-associated kinases (IRAKs), IRAK4, and IRAK1 to the TLR complex (90). Subsequently, IRAK4 and IRAK1 become activated and facilitate the interaction of TRAF6 with the complex (90). This molecular complex then interacts with another complex comprised of TAK1, TAB1 and TAB2, which activates IKK and eventually NF-κB (84, 90). TAK1 activation leads to activates mitogen-associated protein kinase (MAPK) and Janus kinase (JNK), resulting in the upregulation of growth factors, cytokines, chemokines, and cell adhesion molecules (84, 90). Four distinct IRAKs (IRAK-1, IRAK-2, IRAK-M, and IRAK-4) have been identified both in humans and mice (90). Intriguingly, recent reports have documented that IRAK-M functions as a negative regulator of TLR signaling, and IRAK-M knockout mice show an augmented inflammation in numerous organs (92). Numerous studies have investigated the roles of adaptor proteins involved in TLR pathways, including the MyD88-dependent cascade (MyD88 and TIRAP) and the MyD88-independent cascade (TRIF and TRAM) in bacterial infections (84). MyD88 is important for host defense against several bacterial infections, including *S. pneumoniae* (54)*, E. coli* (51)*, K. pneumoniae* (52)*, H. influenzae* (39), *P. aeruginosa* (53), *S. aureus* (53), and *L. pneumophila* (31). However, TIRAP, a molecule upstream of MyD88, is also essential for pulmonary host defense against *E. coli* (56) and *K. pneumoniae* (55). While TRIF plays an essential role in host defense against *E. coli* (51) and *P. aeruginosa* (57) challenge, it has shown MyD88 plays a more dominant role than TRIF during host defense against K*. pneumoniae* (52). This suggests that pathogens can activate both MyD88-dependent and MyD88-independent signaling cascades through distinct bacterial components.

The activation of TLRs and the subsequent cell signaling lead to the production of pro-inflammatory cytokines, including interleukin-6 (IL-6), interleukin-1 (IL-1), and tumor necrosis factor-alpha (TNF-α), which recruit immune cells to the site of infection (93). In addition, TLR activation also leads to the upregulation of numerous cell surface receptors, such as Fcγ receptors, which are responsible for the phagocytosis of opsonized bacteria (90), as well as of antimicrobial peptides and reactive oxygen species, which help to kill bacteria that have been engulfed by phagocytes (90). Furthermore, the activation of TLRs triggers the production of cytokines such as IL-12, which promotes the differentiation of T helper 1 (Th1) cells that are essential for the clearance of intracellular bacterial infections (83). TLRs also cause the upregulation of co-stimulatory molecules on antigen-presenting cells that are necessary for the activation of naive T cells (83, 93). While TLR activation leads to the production of mRNA for pro-IL- 1β and pro-IL-18, a caspase is required to convert these inactive forms of IL-1β and IL-18 into their respective active forms to initiate inflammatory signaling (20). NLRs are intracellular PRRs that play a critical role in innate immune response and host physiology, and their characteristic features are a central NOD (or NACHT) domain, which is necessary for oligomerization, an N-terminal homotypic protein-protein interaction domain and a C-terminal leucine-rich repeats (LRRs) responsible for agonist sensing or ligand binding (94).

### NLRs

The multimeric protein complexes, termed “Inflammasomes”, are formed by some NLRs, such as NLRC4, NLRP3 and NLRP6, and contain an activated caspase that is responsible for converting and activating IL-1β and IL-18 for the initiation of inflammatory signaling (20) (**Table 1**). NLRs can be categorized into three groups according to the phylogenetic structure of their domains: (1) NODs (NOD1-5 and CIITA), (2) the NOD, LRR, and PYD containing (NLRPs) or NALPs (NLRP1–14), and (3) the IPAF (ICE-protease-activating factor) family of NLRs (NLRC4 and NLR family apoptosis inhibitory proteins or NAIPs) (20, 95). NOD1 and NOD2 were the initial NLRs identified as pathogen sensors, and both NOD1 and NOD2 encompass CARD domains at their N terminal domain which are known to signal through the adaptor molecule RIP2 (96). NOD1 has been shown to recognize γ-d-glutamyl-meso-diaminopimelic acid (i.e., DAP), a cell wall component of Gram-negative bacteria, while the NOD2 LRR binds the MDP (muramyl dipeptide) motif present in the Gram-negative and Gram-positive bacterial peptidoglycans (96, 97).

Studies have demonstrated that NOD1 and/or NOD2 are capable of sensing *C. pneumoniae* (59), *S. aureus* (63), *S. pneumoniae* (61, 62, 64), *A. baumannii* (58), and *L. pneumophila* (60, 65) through recognition of their respective peptidoglycan ligands and also by peptidoglycan-independent mechanisms (98). The deficiency of the NOD1 and/or NOD2 gene in mice infected with *C. pneumoniae* (59), *S. aureus* (63), or *L. pneumophila* (60) resulted in attenuated levels of pulmonary cytokines and chemokines with decreased neutrophil infiltration into the lungs. However, the bacterial burden of these deficient mice varied based on the bacterial infection. *C. pneumoniae*-infected NOD1/2 deficient mice had impaired bacterial clearance, while *L. pneumophila*-infected NOD1/2 deficient mice had enhanced pulmonary bacterial burden (59, 60). *S. aureus*-infected WT and NOD2 gene-deficient mice showed no significant difference in pulmonary colony forming units (CFUs) (63).

The most extensively studied NLR is NLRP3. Although NLRP3 has mainly been investigated in human and murine macrophages, it is also expressed in airway epithelial cells of human and murine origin during bacterial infections (96). The defining features of NLRP3 are the N-terminal PYD that homotypically binds the PYD of ASC and the requirement for two discrete signals for activation in the conical pathway (99). The first signal comes from TLR activation which *primes* and induces the expression of NLRP3 through NF-κB activation. Once the amount of NLRP3 in the cytosol reaches the threshold, the second signal originates from one or more PAMPs which results in the assembly of the NLRP3 inflammasome (96, 100). The NLRP3 inflammasome can be activated through TLRs by multiple molecular or cellular events including ionic flux, mitochondrial dysfunction, the production of reactive oxygen species (ROS), and lysosomal damage (101). Though other inflammasomes do not require TLR signaling for the synthesis of their integralcontain molecules, the generation of mature IL-1β by other inflammasomes may be influenced by TLR activation since TLR signaling contributes to the enhanced cytosolic expression of pro–IL-1β (96, 99).

The NLRP3 inflammasome has two additional non-canonical activation pathways including a pathway induced by LPS internalization into the cytoplasm and resulting in pyroptosis, the release of ATP and K+ efflux, which then drive the NLRP3 inflammasome assembly and release of IL-1β (101) as well as an K+ efflux independent pathway that does not induce pyroptosis (101). This second alternative pathway is activated in human monocytes after LPS stimulation and entails receptor-interacting serine/threonine-protein kinase 1 (RIPK1), FAS-mediated death domain protein (FADD), and caspase-8 (101). The PAMPs that activate these various NLRP3 inflammasome pathways include bacterial pore-forming toxins such as α-hemolysin (*S. aureus*) (66, 102), streptolysin O (*Streptococcus pyogenes*) (103), and pneumolysin (*S. pneumoniae*) (67). This pathway culminates in NLRP3-induced IL-1β production in both murine and human macrophages (96). Note that bacterial pore-forming toxins, apart from inducing NLRP3 activation, can also directly induce alveolar-capillary barrier dysfunction by increasing intracellular Calcium (104, 105). NLRP3 activation has also been shown to exert a protective role during *K. pneumoniae* infection by increasing inflammatory cell recruitment and decreasing mortality (68).

A part of the IPAF family of NLRs, NLRC4 has also been reported to play an essential role in innate immune regulation during pulmonary infections. The NLRC4 inflammasome gets activated during *K. pneumoniae* infection even though *K. pneumoniae* does not express either flagellin or a type III secretion system (T3SS or injectosome) (74). NLRC4 has been shown to cooperate with TLR5 to induce protective pulmonary immunity against *P. aeruginosa* (73). We and other researchers have shown that the NLRP6 inflammasome serves as a negative regulator of neutrophil recruitment and function during pulmonary infection with *S. aureus* (75) *and S. pneumoniae* (76). However, we have recently shown that NLRP6 is a positive regulator of neutrophil recruitment and function during *K. pneumoniae*-induced pneumonia-derived sepsis where the NLRP6-deficient mice had reduced survival, increased bacterial burden, and decreased neutrophil migration and function (77). By contrast, a recent investigation has reported NLRP6 to be detrimental during *S. pneumoniae* pulmonary infection (106).

Our previous studies have shown host survival and bacterial clearance is dependent on NLRP12 activation following *K. pneumoniae* infection (78). The absent in melanoma 2 (AIM2) macromolecular inflammasome complex forms in response to cytosolic double-stranded DNA (dsDNA) which leads to pyroptosis and the maturation of proinflammatory cytokines IL-18 and IL-1β (107). Regardless of the sequence, the sugar-phosphate backbone of dsDNA binds to the HIN domain of AIM2 and relieves the PYD for self-oligomerization, and the PYD interaction with ASC results in the activation of the AIM2 inflammasome (108). A recent study has shown the AIM2 inflammasome is required for host defense against *S. pneumoniae* pulmonary infection by inducing IL-1β maturation and secretion in macrophages (81). The recruitment of ASC is required for the formation of inflammasomes, and studies have shown the individual deletion of ASC during *P. aeruginosa* (79) and influenza with *S. aureus* co-infection (80) modulates the immune response. Though inflammasomes are vital components of the innate immune system during responses to several pathogens, there have been several studies that have shown extracellular bacteria activating the NLRs, such as NLRC4, NLRP6, and/or NLRP3, to induce pyroptosis and cause detrimental inflammatory-induced damage in the host (20).

## Pulmonary viral infection

Respiratory infections commonly consist of multiple different pathogens, and post-influenza bacterial pneumonia is a main cause of mortality and morbidity during both seasonal and pandemic influenza virus infections (109) (**Table 1**). With the most recent SARS-CoV pandemic, there has been more interest and research into viral respiratory infections and diseases. SARS-CoV induces severe acute respiratory syndrome (SARS) characterized by excessive lower respiratory tract infection. Severe acute respiratory syndrome coronavirus 2 (SARS-CoV-2) is the cause of COVID-19 and the global pandemic in 2020 (110). By the end of 2021, over 287 million cases were reported worldwide with over 5.4 million deaths, and in the United States, more than 54.5 million confirmed cases and more than 825,000 deaths were documented (111).

While the impact of SARS-CoV2 has brought attention to the severity of respiratory viral infections, other respiratory viruses are still clinically relevant as they also can cause respiratory distress and exacerbate other diseases and disorders. Other prominent respiratory viruses include influenza and human rhinovirus (RV), both single-stranded RNA (ssRNA) viruses. Respiratory tract infections caused by influenza kill up to 500,000 people and cost up to $167 billion annually for treatment and care (4). Influenza pandemics have shown the severity of viral-bacterial coinfections, with the deaths among patients with the Spanish flu (caused by an H1N1 influenza virus) predominantly caused by secondary bacterial infections (112). During the most recent H1N1 pandemic, *S. pneumoniae* was found to be the most common coinfection contributor, but there was no significant association between bacterial coinfection and ICU mortality (112, 113). RV, the most common viral infectious agent in humans, circulates worldwide and is responsible for more than 50% of cold-like illnesses, costing billions of dollars annually in medical visits (114). Though RV infections can result in mild symptoms, RV has been shown as the most common respiratory viruses detected in patients with otitis media, bronchiolitis, croup, and pneumonia, and is known as a common exacerbator of chronic lung diseases (115).

Asthma exacerbations remain a major cause of disease morbidity and a significant financial burden to patients (116). The frequent triggers of asthma exacerbation are viral respiratory infections such as RV, influenza, and coronaviruses (116). In both children and adults, hospital admissions for asthma exacerbations correlate with the seasonal increase in RV infections (116). During the H1N1 influenza A pandemic in 2009, the mortality and admission rate to the intensive care unit with H1N1 infections often correlated with asthma exacerbation (116). Respiratory infections usually consist of multiple different pathogens. The influenza pandemics and the most recent SARS-CoV pandemic have underscored the clinical relevance of viral-bacterial coinfections. Although *S. pneumoniae*/H1N1 co-infections did not significantly increase mortality, there are reports that SARS-CoV2/*S. pneumoniae* co-infections increase mortality 7-fold (117).

### TLRs

Following viral infection, the host triggers a rapid innate response characterized by the production of IFNs and inflammatory cytokines/chemokines to inhibit virus replication and destroy the invading virus (118). Upstream of this response, certain TLRs are responsible for the recognition of viral PAMPs, including viral nucleic acids and viral proteins, which results in the activation of numerous intracellular signaling cascades that lead to antiviral IFN and inflammatory cytokine response (118). On the host cell membrane, TLR10 can recognize extracellular bacterial and viral proteins (119). Endosomal TLRs, within endosomes and lysosomes, encounter and bind nucleic acids released from engulfed microbes, including viruses (120). TLR3 is known to recognize double-stranded RNA produced by viruses during replication, while TLR9 recognizes unmethylated DNA with cytosine-phosphate-guanosine (CpG) motifs that are commonly found in bacterial and viral DNA (88, 121). Though TLR3 has not been shown to affect the clearance of respiratory syncytial virus (RSV), an ssRNA virus, TLR3 was shown to alter cytokine concentration and mucus production in the lungs after RSV infection (38). Studies have also shown TLR3 activation during influenza A virus (IAV)-induced acute pneumonia to be detrimental to mice, resulting in decreased survival, viral clearance, and recruitment of neutrophils to the lungs (37). However, TLR2/6 and TLR9 have been shown to work synergistically to protect mice during lethal IAV-induced pneumonia (122).

Both TLR7 and TLR8 recognize single-stranded RNA produced by viruses during replication (123, 124). However, recent studies have shown activation of TLR7 or TLR8 triggers distinct IRF and NF-κB pathways to induce differential cytokine/chemokine profiles to promote inflammation (123, 125). IAV infection of TLR7-deficient mice resulted in increased morbidity and neutrophil influx; however, lung viral titers were similar to that seen in WT mice (44). TLR7 was also shown to recognize RSV and initiate an innate immune response (45). Though studies have shown TLR8 is activated during influenza and RSV infections, there is no current knockout (KO) mouse model available to differentially observe TLR8’s role during these infections (46, 47). TLRs recognize viral RNA and DNA in endosomes, while RIG-I-like receptors (RLR) are the main PRRs that recognize cytoplasmic viral RNA (126). The activation of these receptors primarily leads to the activation of an antiviral innate immune response through the production of IFNs (126).

### NLRs

Viruses can trigger the activation of the NLRP3 inflammasome, a complex of proteins involved in the immune response (21). Mitochondrial antiviral signaling proteins, also known as IPS-1/cardif/VISA, located in the outer membrane of mitochondria, can activate the NLRP3 inflammasome, leading to downstream signaling. Viral RNA and a non-structural protein called PB1-F2 have also been implicated in inflammasome activation (21, 127). PB1-F2 can further activate the release of IL-1β by inducing aggregation in phagosomes (21, 127). In another report, expression of the influenza virus M2 protein, a proton-specific ion channel, in the Golgi apparatus was found to induce NLRP3 inflammasome activation (69, 127, 128). ATP and ATP-dependent K+ efflux, both of which can cause NLRP3 activation, have been associated with several viruses, including RSV and influenza virus infections (21, 129). It has been suggested that ATP released from dead cells during influenza virus infection can induce NLRP3 activation. In another study, it was found that genomic influenza RNA was unable to cause an inflammasome response in the absence of ATP in macrophages derived from bone marrow (130). The role of ATP-related NLRP3 activation in influenza infection was demonstrated in cocultures of macrophages and epithelial cells, and it was also shown that ATP signaling through the P2X7 receptor is important for NLRP3 activation *in vivo* (21, 34, 129).

The activation of NLRP3 and NLRC5, along with caspase-1 maturation and IL-1β release, is also induced by viroporin 2B of human rhinovirus (70). This cytotoxic pore-forming protein is thought to control ion channel activity, resulting in an influx of cytosolic Ca2+ from the Golgi and endoplasmic reticulum, leading to inflammasome activation (21, 130). The overlapping activation of NLRP3 and NLRC5 by the same pathogen and their similar responses to human rhinovirus infection indicate a heterogeneous inflammasome or cooperative interaction between these two inflammasomes (21, 131, 132). Similarly, human RSV signals through its small hydrophobic viroporin molecule, which results in caspase-1 activation and IL-1β maturation (133). The ion channel activity of viroporin disturbs the intracellular ion balance, leading to NLRP3 inflammasome activation (134). Following RSV infection, pro-IL-1β synthesis is caused by the TLR2/MyD88/NF-κB pathway, along with K+ efflux and ROS generation, resulting in the establishment of the NLRP3 inflammasome (132). Both signals ultimately lead to the maturation and activation of caspase-1 and the release of IL-1β. This was validated by the lack of inflammasome activation in RSV mutants lacking viroporin, as well as through use of lipid raft disruptors and viral ion channel-inhibiting drugs (131). AIM2-dependent IL-1β secretion from macrophages was shown during influenza A infection, and upregulation of the inflammasome-related AIM2 gene was shown during asthma exacerbation by rhinovirus-A16, though very little is known about the AIM2 function during rhinovirus infection (135). While the mechanism associated with inflammasome activation by SARS-CoV infection is not completely understood, it has been associated with the uncontrolled release of proinflammatory cytokines, such as MCP-1, IL-6, IL-18, IFN-γ, and IL-1β in the lungs, blood, and lymph nodes (134, 136). In addition, N protein from SARS-CoV augments NLRP3 activation in order to induce inflammation in immune cells and the lung (137).

## Pulmonary fungal infection

Fungal infections pose a significant threat, particularly to individuals that are immunocompromised (21, 138). *Aspergillus fumigatus* and *Cryptococcus neoformans* are common causes of fungal infections that can result in life-threatening conditions, particularly in immunocompromised individuals and organ transplant recipients (138, 139). Another significant fungal infection is Paracoccidioidomycosis (PCM), an endemic disease caused by *P. brasiliensis* that is responsible for systemic granulomatous mycosis, which is commonly found in specific Latin American countries such as Brazil, Argentina, Venezuela, and Colombia (140).

### TLRs

Respiratory fungal infections have been reported to activate TLR2, TLR4, TLR9, and NLRP3 (89, 97, 141) (**Table 1**). Studies have shown that TLR2 and MyD88 are required for protection during *C. neoformans* infection (142–144). In human host cells, TLR2-, TLR4- and MyD88-dependent activation were reported to play a critical role in cytokine production, polymorphonuclear neutrophil (PMN) activation, and vulnerability to infection by *A. fumigatus* (35, 142, 145, 146). Conidia, a spore produced by various fungi, are recognized by TLR2 and TLR4 and result in the production of proinflammatory cytokines, while the hyphae of the fungi are recognized by TLR2 and stimulate IL-10 production (142, 147, 148). The germination from conidia to hyphae was speculated as an escape mechanism for *Candida albicans* and *A. fumigatus* (147, 148). For instance, the ligands for TLR4 are present on *A. fumigatus* conidia but not hyphae, while the ligands for TLR2 are on *A. fumigatus* conidia and hyphae (148, 149). TLR4 is also activated by the binding of the O-linked mannans from *C. albicans*, as well as glucuronoxylomannan (GXM) from *C. neoformans* (148–150). There have been reports the MyD88 adaptor protein plays a role in cell signaling and protective responses during fungal infection, while other reports have shown MyD88 signaling and activation of NF-κB to be insignificant for fungal clearance (35, 142, 145, 151, 152). TLR2 was shown to recognize fungal β-glucans of fungal species, and to specifically interact with phospholipomannans (PLMs), linear beta-1,2-oligomannoside structures unique to *C. albicans* (149). TLR2 can also form TLR2/TLR1 and TLR2/TLR6 heterodimers to recognize the GXM component of *Cryptococcus neoformans* (149, 153). *A. fumigatus* was found to activate mouse TLR2/6 heterodimers but not that of humans, whereas both human and mouse TLR2/1 heterodimers recognize *A. fumigatus* (149, 154).

### NLRs

The role of the NLRP3 inflammasome in defending against various fungal infections, such as the ones mentioned above, has been extensively studied, but the functions of other inflammasomes, including NLRP6, NLRP12 and NLRC4, are still not well understood. In human monocyte cell lines, activation of the NLRP3 inflammasome and subsequent processing of IL-1β are triggered by hyphal fragments of *A. fumigatus*, and both processes rely on the activity of Syk tyrosine kinase (82, 139, 155). Mice with a double deficiency in both NLRP3 and AIM2 exhibited increased vulnerability to pulmonary *A. fumigatus* infections when compared to WT mice, while mice lacking only the inflammasome displayed a phenotype similar to that of WT mice (82). Furthermore, NLRP3- and AIM2-mediated secretion of IL-1β and IL-18 was found to be important for conferring protection against *A. fumigatus* in an immunocompromised mouse model (82). On the contrary, an independent study utilizing mice with NLRP3 deficiency showed enhanced host protection and decreased fungal burden in the lungs following infection. Interestingly, NLRP3 KO mice exhibited increased susceptibility to *A. fumigatus* compared to WT mice when exposed to a higher dose of *A. fumigatus* (71). *C. neoformans* is another type of opportunistic fungal pathogen that commonly infects individuals with compromised immune systems (138). Studies conducted using human macrophages and mouse models have revealed that the NLRP3 inflammasome is triggered by capsular *C. neoformans* infection (72). In mouse dendritic cells, the secretion of IL-1β in response to *C. neoformans* necessitates NLRP3 activation, while NLRC4 or AIM2 inflammasomes are not involved (72). In an *in vivo* setting, proper recruitment of neutrophils and clearance of the fungus from the lungs was found to depend on NLRP3 activation (72, 138). However, another study demonstrated that internalized, encapsulated *C. neoformans* can cause not only canonical caspase-1 but also noncanonical caspase-8 inflammasome activation in mouse dendritic cells (71, 138, 139).

## Concluding remarks

Respiratory diseases are major public health threats worldwide, and the overuse of antibiotics and antivirals to treat these infections pressures these pathogens to gain antibiotic or antiviral resistance. The rise of these antibiotics or antiviral-resistant strains has caused severe illnesses and has given precedence to the development of other treatment methods and therapies. The innate immune response is essential for the effective elimination and control of infections, and the modulation of precise aspects of the innate immune response has become a popular target for immunomodulatory therapeutics. The experimental evidence reported highlights the relevance of TLR and NLR activation in response to pathogens and the corresponding inflammatory response. It is now clear that TLRs and NLRs not only control innate immune responses, but also trigger adaptive immune responses. Of note, the interaction between the host and pathogen in the lung decides if the activation of TLRs and NLRs by infectious agents is protective or detrimental. Nonetheless, the TLR and NLR pathways are complex, and it is possible that crosstalk occurs within TLR and NLR cascades and between TLR and NLR pathways. Therefore, further studies are necessary to better identify the specific mechanisms and pathways mediated by TLRs and NLRs for the expression, activation, and regulation of respiratory innate defense against microbial infections.

Despite the importance of NLRs in bacterial, viral, and fungal infections in the lung, much remains to be learned, as new NLRs, their ligands, and signaling pathways are being discovered. Although understanding of innate immune defense has improved, future challenges will be to apply knowledge of innate immune defense to design host-targeted immunotherapies to mitigate excessive inflammation-mediated tissue damage following microbial infection in the lung while controlling microbial growth and multiplication. In this context, several TLR agonists and antagonists have shown promise in preclinical animal models and have now entered clinical research. Moreover, downstream NLR molecules, such as caspase-1, IL-1 receptor antagonists and IL-1β, have also been evaluated in preclinical models.

## Author contributions

Wrote the draft: JL and YK. Edited the draft: SJ. All authors contributed to the article and approved the submitted version.
